# Comparative Study on the Nutritional, Textural and Flavor Profiles of Mandarin Fish (*Siniperca chuatsi*) in Industrialized Recirculating and Traditional Pond Aquaculture Systems

**DOI:** 10.3390/foods14234028

**Published:** 2025-11-24

**Authors:** Weifa Su, Rongfeng Wu, Hongjie Fan, Gaohua Yao, Wei Liu, Shimi Li, Ningyu Zhu, Qianrong Liang, Xueyan Ding, Bin Zheng, Xingwei Xiang, Fan Zhou

**Affiliations:** 1 College of Food Science and Technology, Zhejiang University of Technology, Hangzhou 310014, China; suweifa@zjut.edu.cn (W.S.); 211124260009@zjut.edu.cn (H.F.); zhengbin@zjut.edu.cn (B.Z.); 2 Huzhou Wushi Ecological Agriculture Co., Ltd., Huzhou 313002, China; 15868206888@163.com; 3 Zhejiang Fisheries Technical Extension Center, Hangzhou 310012, China; 15988855806@163.com (G.Y.); zjscliuwei@163.com (W.L.); lisimin1027@163.com (S.L.); zny1984@sina.com (N.Z.); lqianr1990@163.com (Q.L.); dingxy_zjfish@126.com (X.D.)

**Keywords:** *Siniperca chuatsi*, texture properties, nutritional composition, flavor compounds, gut microbiota, muscle quality, GC-IMS

## Abstract

Mandarin fish (*Siniperca chuatsi*) is a highly valued freshwater species in China, owing to its high-quality meat and economic importance. This study comparatively evaluated the effects of an industrialized recirculating aquaculture system (RAS) and traditional pond aquaculture system (TPAS) on the muscle quality and further explored the role of gut microbiota in muscle quality regulation. Our results showed that the RAS resulted in superior textural properties, with meat that was significantly more tender and elastic. The RAS also promoted higher muscle protein and reduced lipid levels. Notably, the RAS elevated sweet-tasting amino acids (Gly and Pro) while suppressing bitter amino acids (His). Electronic nose and GC-iMS analyses revealed distinct flavor compound profiles between the two systems, and the RAS enriched desirable volatiles (esters and alcohols) while suppressing aldehydes (e.g., nonanal and heptanal) associated with off-flavors. Gut microbiota profiling indicated higher diversity and enriched beneficial genera (e.g., *Cetobacterium*, *Lactobacillus*) in RAS-treated fish. We found that the *Cetobacterium* in the RAS group showed a significant positive correlation with sweet amino acids and pleasant flavor substances (such as esters, alcohols), while exhibiting a negative correlation with undesirable flavor precursors (such as certain aldehydes). This finding contributes to the sustainable and high-efficiency advancement of intensive *Siniperca chuatsi* aquaculture.

## 1. Introduction

Mandarin fish (*Siniperca chuatsi*) is a highly valued freshwater species in China, owing to its high-quality meat and economic importance. It is widely distributed in China, with Hubei, Hunan, Anhui, Zhejiang and other regions being its main producing areas. Mandarin fish has plump and tender flesh, a delicious taste, and few bones. It is highly prized for its rich nutritional value, featuring an abundance of high-quality protein, essential amino acids, and a beneficial fatty acid profile, coupled with a relatively low fat content, thus being praised as “a top-grade fish and a delicacy on the banquet”. It is also known as one of the “Four Famous Freshwater Fishes” in China, along with the Yellow River carp, Songjiang perch, and Xingkai Lake whitefish [1]. With the improvement of people’s living standards and the increasing demand for high-quality aquatic products, mandarin fish is favored on the market due to its unique advantages, and the market demand continues to rise [2]. In 2024, China produced 0.47 million tons of mandarin fish, generating over USD 2.8 billion in output value [3].

In the field of mandarin fish aquaculture, the traditional pond culture system is relatively common, but it faces many challenges. For example, it is difficult to regulate water quality, which can easily lead to diseases, thereby affecting the growth rate and muscle quality of mandarin fish [4]. To overcome these difficulties, new aquaculture systems have been emerging. The industrial recirculating aquaculture system realizes zero-pollution discharge of aquaculture tail water by incorporating tail water purification devices and applying biological sewage discharge technology [5,6]. At the same time, it uses an intelligent monitoring system to regulate key parameters in real time, such as water temperature and dissolved oxygen, simulating natural water quality and creating an excellent growth environment for mandarin fish. Land-based circular bucket recirculating aquaculture technology, combined with the Internet of Things intelligent technology, not only improves the utilization rate of aquaculture space but also enables precise control of the aquaculture process. However, the differential impacts of these systems on mandarin fish growth performance, muscle quality, and economic viability remain inadequately characterized.

A large number of previous studies have shown that aquaculture systems have an important impact on fish growth, muscle quality, and nutritional components. For instance, distinct systems induce significant variations in muscle fatty acid composition, amino acid profiles, and volatile flavor compounds in largemouth bass (*Micropterus salmoides*), directly linking system design to muscle quality [7]. Furthermore, gut microbiota has been established as a key regulator of muscle quality in farmed fish [8]. Despite these insights, the interplay between aquaculture systems, gut microbiota, and muscle quality in mandarin fish remains poorly understood and needs further investigation.

Therefore, this study aimed to comparatively assess the effects of traditional pond (TPAS) and industrialized recirculating (RAS) aquaculture systems on the muscle quality of mandarin fish. We hypothesized that RAS rearing would promote superior muscle texture, nutritional composition, and flavor characteristics, and that these improvements would be associated with distinct gut microbiota profiles and functional potentials. These quality parameters were further investigated for their correlations with gut microbiota profiles.

## 2. Materials and Methods

### 2.1. Experimental Materials

Both the pond-cultured mandarin fish and the recirculating aquaculture system (RAS)-cultured mandarin fish used in this study were collected from an aquaculture farm in Huzhou city, Zhejiang Province, China. In the TPAS, mandarin fish were stocked at a density of 2 fish per cubic meter and reared in an earthen pond relying on natural water, with the temperature fluctuating seasonally between 18 and 30 °C. In contrast, the RAS maintained a high-density culture of 20 fish per cubic meter within an indoor, fully controlled environment. The water quality in the RAS was rigorously managed: temperature was kept constant at 25–28 °C, with dissolved oxygen above 5 mg/L, pH between 7.2 and 8.3, and ammonia nitrogen and nitrite levels controlled below 2.0 mg/L and 1.0 mg/L, respectively. In both systems, fish were fed to apparent saturation twice daily (at 6:00–7:00 and 17:00–18:00) with a commercial extruded compound feed formulated specifically for mandarin fish. The mandarin fish samples used in this study were collected in April 2025. The fish in the TPAS group had an average body weight of 532.6 ± 68.3 g and an average body length of 28.5 ± 1.8 cm, whereas those in the RAS group had an average body weight of 568.9 ± 72.1 g and an average body length of 29.3 ± 1.5 cm. All experimental fish had reached sexually mature, marketable size.

The experiment was designed with three independent culture units as biological replicates for each system. From each unit, ten healthy adult mandarin fish of uniform size were randomly selected, resulting in a total of thirty fish per system. Mandarin fish were euthanized by delivering a quick, forceful blow to the head with a blunt object. All samples were processed within 1 h post-euthanasia at ambient temperature (25 °C). To ensure consistency, muscle samples for all subsequent analyses were systematically collected from the same anatomical location: the dorsal epaxial muscle on both sides of each fish. The moisture content and textural properties of the fish meat were measured within 24 h. Intestinal and meat samples, including those for analyzing proximate composition, free amino acids, and volatile flavor compounds, were immediately stored at −20 °C. In contrast, samples dedicated to gut microbiota analysis were promptly stored at −80 °C for further analysis.

Ethical statement: All animal experimental protocols were approved by the Institutional Animal Care and Use Committee of Zhejiang Fishery Technology Extension Center, Hangzhou, China (approval number: SYXK-ZHE-2020-0009, approval date: 26 May 2020), and were performed in accordance with the Animals (Scientific Procedures) Act 1986, its associated guidelines, and EU Directive 2010/63/EU on the protection of animals used for scientific purposes.

### 2.2. Determination of Physical Properties

In this experiment, a Rapid TA texture analyzer (Shanghai Tengba Instrument Science and Technology Co., Ltd., Shanghai, China; the test probe is a P/36 column probe) was utilized for the texture profile analysis of mandarin fish to determine the hardness, elasticity, cohesion, mastication, and adhesion of muscles.

### 2.3. Determination of Nutrient Content

For moisture determination, the GB 5009.3-2016 standard was followed [9], baking samples in an oven (DHG-9070A, Yiheng Technical Co., Ltd., Shanghai, China) at 105 °C to constant mass. Crude protein was quantified using the Kjeldahl method GB 5009.5-2016 [10] with a K9860 automatic Kjeldahl analyzer (Hanon Advanced Technology Group Co., Ltd., Jinan, China). Crude ash content was determined according to GB 5009.4-2016 [11] using a muffle furnace (SX2-4-10, Jingke Experimental Equipment Co., Ltd., Shanghai, China) at 550 °C. Crude fat was analyzed via the Soxhlet extraction method GB 5009.6-2016 [12] with an SXT-06 Soxhlet extraction system (Shanghai Xiande Experimental Equipment Co., Ltd., Shanghai, China).

### 2.4. Free Amino Acid Content

The determination of free amino acid content was performed following the method described by Xu et al. [13], with minor modifications. For amino acid hydrolysis, 2 g of fish meat and 15 mL of 15% trichloroacetic acid (TCA; Hangzhou Shuangmu Chemical Co., Ltd., Hangzhou, China) solution were mixed in a 50 mL centrifuge tube. The mixture was homogenized for 10 s and then left to stand for 2 h. It was subsequently centrifuged at 10,000 rpm for 15 min using a centrifuge (ST16R, Thermo Fisher Scientific, Hangzhou, China). After centrifugation, 10 mL of the supernatant was taken, and its pH was adjusted to 2.0 with a 1 mol/L NaOH solution (Sinopharm Chemical Reagent Co., Ltd., Shanghai, China). The volume was then made up to 25 mL, and the solution was filtered through a 0.22 μM organic microporous filter membrane prior to analysis using an automatic amino acid analyzer (LA8080, Hitachi Ltd., Tokyo, Japan). Each group of samples was assayed at least 6 times, and the mean value was calculated.

### 2.5. Determination of Volatile Metabolites

Analysis of volatile organic compounds (VOCs) was performed using headspace gas chromatography–ion mobility spectrometry (HS-GC-IMS; FlavourSpec instrument, G.A.S. Gesellschaft für analytische Sensorsysteme mbH, Dortmund, Germany) [14]. Following incubation at 65 °C for 20 min, 500 μL of headspace sample was injected into an FS-SE-54-CB-1 capillary column (15 m × 0.53 mm, 1 μm film thickness) using a syringe preheated to 85 °C. High-purity nitrogen (99.99%) served as both carrier and drift gas. The programmed gas flow rate was as follows: initial hold at 2 mL/min for 2 min, followed by a linear ramp to 15 mL/min over 10 min, then to 100 mL/min over 20 min, and finally to 150 mL/min over 30 min. Data represent the mean values from three replicate analyses. IMS data were processed using multiple software packages (LAV v.2.0.0; G.A.S. Gesellschaft für analytische Sensorsysteme mbH, Dortmund, Germany; including Reporter, Gallery Plot, GC-IMS Library Search) for comprehensive analysis. VOC identification involved the NIST library and G.A.S. IMS database search software, combined with retention indices and drift times.

### 2.6. Determination of Electronic Nose

Electronic nose testing methods and parameters were consistent with published work [15], with minor adaptations. This system assessed the impact of different aquaculture systems on mandarin fish muscle flavor. Its sensor array comprised 18 metal oxide sensors. Sample processing and analysis were based on the ratio of sensor resistance (G) for sample headspace volatiles to that (G0) for standard gas. Each distinct fish sample underwent six repetitive analyses.

### 2.7. Gut Microbiota Analysis

Total genomic DNA was extracted from the sedimented samples following the manufacturer’s protocol for the QIAamp^®^ DNA Stool Mini kit (QIAGEN, Hilden, Germany). DNA quality assessment and concentration quantification were conducted using a NanoDrop 2000 spectrophotometer (Thermo Fisher Scientific, Wilmington, DE, USA). Amplification of the bacterial 16S rRNA gene V3–V4 variable regions utilized primers 338F (5′-ACTCCTACGGGAGGCAGCAG-3′) and 806R (5′-GGACTACHVGGGTWTCTAAT-3′), with the PCR cycling protocol including 95 °C for 30 s, 55 °C for 30 s, and 72 °C for 45 s (27 cycles). The 20 μL reaction mixture consisted of 4 μL 5 × TransStart FastPfu buffer, 2 μL of 2.5 mM dNTPs, 0.8 μL of each primer (5 μM), 0.4 μL TransStart FastPfu DNA polymerase, 10 ng DNA template, and doubly distilled H_2_O. Verification of amplicon size was achieved through 1% agarose gel electrophoresis. Subsequent paired-end sequencing (PE300) of amplicons was performed on the Illumina MiSeq platform (Illumina, San Diego, CA, USA) by Majorbio Bio-Pharm Technology Co., Ltd. (Shanghai, China).

Post-demultiplexing, sequence merging was executed with FLASH (v1.2.11), followed by quality filtering using fastp (v0.19.6). High-quality sequences underwent denoising via the DADA2 plugin within the Qiime2 (v2020.2) pipeline using default parameters, producing single-nucleotide resolution data based on sample-specific error models. The DADA2-processed output represented amplicon sequence variants (ASVs). To standardize sequencing depth for alpha and beta diversity analyses, sequences per sample were subsampled at 4000, achieving an average Good’s coverage of 97.90%. Taxonomic assignment of ASVs was carried out in Qiime2 by employing the Vsearch consensus classifier against the SILVA 16S rRNA database (v138). All 16S rRNA microbiome sequencing data analyses were performed on the Majorbio Cloud Platform (https://www.majorbio.com (accessed on 1 August 2025)).

### 2.8. Statistical Analysis

**Figure 1:**
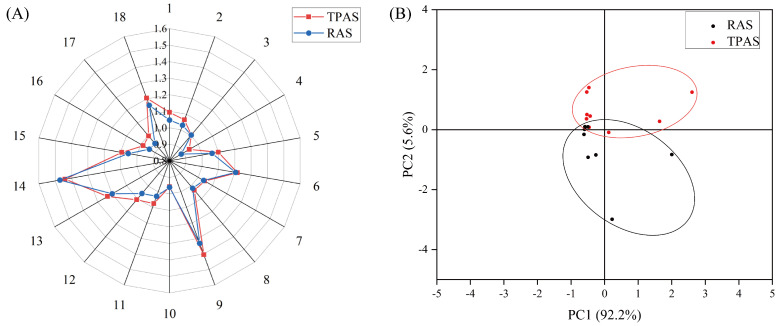
Radar chart of the electronic nose response values (**A**) and principal component analysis score plot (**B**).

All experimental data are presented as the mean ± standard deviation (mean ± S.D.). Prior to one-way analysis of variance (one-way ANOVA), all data were subjected to normality assessment via the Shapiro–Wilk test and homogeneity of variance assessment via the Levene test using SPSS 23.0 software (IBM Corporation, Armonk, NY, USA, *p* > 0.05). Subsequently, one-way ANOVA and Duncan’s multiple range test were performed with SPSS 23.0, and a *p*-value of less than 0.05 (*p* < 0.05) indicates a statistically significant difference. Figure 1 was plotted using Origin Pro 2021 (Origin Lab Corp., Northampton, MA, USA). The corresponding bioinformatics charts were created using the Majorbio website tool. For the analysis of high-dimensional omics data, all statistical computations were performed on the Majorbio Cloud Platform. For gut microbiota, alpha diversity (Chao1, Shannon) and beta diversity (based on unweighted UniFrac distances) were calculated, and the Linear Discriminant Analysis Effect Size (LEfSe) method was applied to identify differentially abundant taxa, using an LDA score threshold of >3.0 and a *p*-value < 0.05 from the Kruskal–Wallis test. Functional and phenotypic profiles were inferred from the 16S rRNA sequencing data; phenotypic predictions (e.g., potential pathogenicity and stress tolerance) were performed using BugBase, while metabolic pathway analysis was predicted (e.g., via PICRUSt2) with KEGG annotation on the Majorbio Cloud Platform. Furthermore, Spearman’s rank correlation analysis was used to investigate associations between significantly altered gut microbiota (genus level), differential metabolites, and key muscle quality parameters, with the false discovery rate (FDR) correction applied for multiple comparisons where appropriate.

## 3. Results and Discussion

### 3.1. Texture Analysis

Texture profile attributes, including hardness, springiness, cohesiveness, gumminess, and chewiness, constitute critical indicators for assessing aquatic product quality. As presented in Table 1, significant differences (*p* < 0.05) were observed in hardness and gumminess between the two aquaculture systems; hardness, defined as the force required to induce compressive deformation in fish muscle and typically associated with superior quality, was significantly lower in recirculating aquaculture system (RAS)-cultured mandarin fish compared to pond-cultured specimens (*p* < 0.05). While no significant differences were detected in springiness, cohesiveness, or resilience, the RAS group showed significantly lower values in mastication and adhesion, parameters respectively associated with reduced chewing effort and less surface stickiness. In general, lower values in these texture parameters contribute to better sensory mouthfeel [16]. Previous studies have shown that a stable environment and proper nutrition in RAS help improve the muscle structure of fish and optimize the composition and proportion of muscle proteins and fats, thereby maintaining the tenderness of fish meat [17]. This is consistent with the findings of our study. Collectively, these results demonstrate superior organoleptic quality in RAS-cultured mandarin fish muscle relative to their pond-cultured counterparts.

### 3.2. Proximate Composition Analysis

As shown in Table 2, the moisture and ash content in the muscle of mandarin fish showed no significant difference (*p* > 0.05) between the two aquaculture systems. However, significant differences (*p* < 0.05) were observed in crude fat and crude protein content. The crude fat content was significantly higher in pond-cultured fish, whereas the crude protein content was significantly higher in RAS-cultured fish (*p* < 0.05). The crude fat content in the muscle of mandarin fish cultured in ponds was significantly higher, which may be related to the higher density of forage organisms, relatively lower exercise intensity, and the system of energy metabolism in their rearing environment, leading to greater fat accumulation [18]. In contrast, the crude protein content of mandarin fish in the RAS group was significantly higher. The RAS environment typically features more controllable water quality parameters (such as dissolved oxygen, temperature, and ammonia nitrogen), lower environmental stress, and optimized feeding strategies. These factors may promote protein synthesis and deposition while reducing the conversion of energy into fat. This result is consistent with multiple studies [17,19], indicating that RAS cultivation helps improve the protein retention efficiency of fish and enhances the nutritional quality of their muscle. In summary, the RAS model exhibits advantages in increasing the muscle protein content of mandarin fish, making it more in line with the healthy dietary demand for high protein and low fat. In contrast, mandarin fish cultured in ponds have a higher fat content, which may affect their textural and flavor characteristics to a certain extent.

### 3.3. Free Amino Acids Analysis

Free amino acids (FAAs) are important flavor compounds and taste components in aquatic products, significantly influencing the taste quality of fish muscle [20]. A total of 16 free amino acids were detected in the muscle of mandarin fish under two aquaculture systems, classified into umami, sweet, and bitter amino acids based on taste characteristics (Table 3). In terms of total content, sweet amino acids were dominant in both groups, followed by bitter and umami types. Although the total umami amino acids were higher in the pond-cultured group (*p* < 0.05), the primary contributor was glutamic acid (Glu), which exceeded its taste threshold in both groups and was significantly higher in the pond-cultured group. Aspartic acid (Asp) remained far below its threshold and contributed minimally to taste.

Notably, the RAS-cultured group demonstrated superior flavor potential and nutritional value in several aspects. Although its total free amino acids (TAA) and total umami amino acids were lower than those in the pond-cultured group, the contents of key sweet amino acids—glycine (Gly) and proline (Pro)—were significantly higher (*p* < 0.05). These amino acids not only provide a mild sweet flavor but are also closely associated with collagen synthesis, which can improve fish meat texture and chewiness [21]. Furthermore, the content of histidine (His)—which is linked to undesirable sour/bitter off-flavors [22]—was significantly higher in the pond-cultured group (*p* < 0.05), whereas it remained relatively low in the RAS. This indicates better flavor purity in RAS-cultured fish. Although the total essential amino acids (EAAs) were higher in the pond-cultured group, the EAA/TAA ratio showed no significant difference in the RAS group, suggesting that overall protein quality was not compromised. Combined with the higher crude protein content reported earlier, these results indicate that the RAS enhances muscle protein deposition while optimizing the composition of flavor-related amino acids. In summary, although the RAS aquaculture system slightly reduced the content of some umami amino acids, it improved the overall flavor profile by increasing key sweet amino acids and reducing off-flavor amino acids. RAS-cultured fish better meet modern consumer demands for aquatic products with high protein, low fat, and pure flavor, demonstrating greater potential for comprehensive quality.

### 3.4. Electronic Nose Analysis

Mandarin fish samples were analyzed using an electronic nose (E-nose), followed by radar chart and principal component analysis (PCA). The E-nose, combining specific sensors with a pattern recognition system, rapidly captures the overall odor profile and accurately characterizes odor features [23]. E-nose response values for muscle odor were measured at different temporary rearing times. Radar chart analysis (Figure 1) indicated that sensors 6, 9, 13, and 14 showed higher responses, suggesting that alkanes, aromatics, alcohols, and aldehydes play a major role in the overall aroma profile. PCA (Figure 1) revealed distinct clustering of aroma components between the two aquaculture systems, indicating significant differences in muscle aroma. The cumulative contribution rate of PC1 and PC2 was 97.8% (>80%), demonstrating that PCA effectively represents the overall sample aroma [24]. While the E-nose discerns overall flavor characteristics, it cannot identify specific changes in individual flavor compounds. Consequently, volatile flavor compounds were further identified and analyzed using Gas Chromatography–Ion Mobility Spectrometry (GC-IMS), building upon the E-nose assessment of the overall aroma.

### 3.5. GC-iMS Analysis

**Figure 2:**
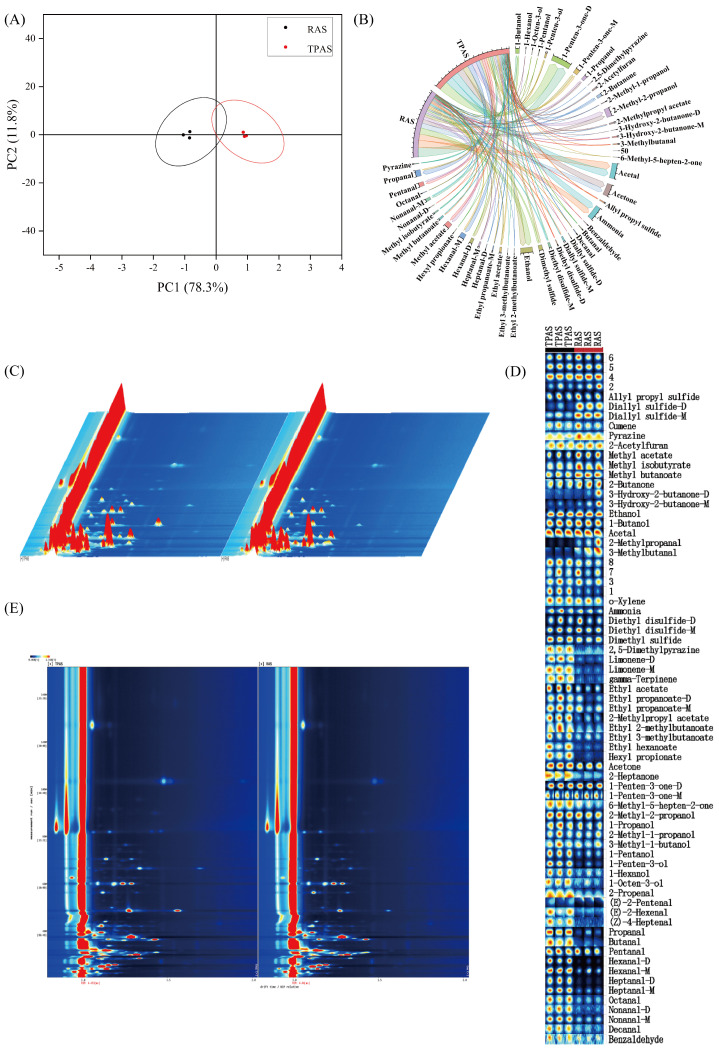
Principal component analysis (PCA) score plot (**A**), chord chart of the proportion of volatile components (**B**), GC-IMS 3D spectrum of volatile components (**C**), gallery plot of volatile components (**D**), and GC-IMS 2D spectrum of volatile components (**E**).

Qualitative analysis identified 54 volatile organic compounds (VOCs) in mandarin fish (*Siniperca chuatsi*) muscle samples, categorized into 8 alcohols, 11 aldehydes, 5 sulfides, 5 alkenes, 3 esters, 4 ketones, pyrazine, ammonia, and heterocyclic compounds (e.g., furans). Volatile flavor compounds were detected using Headspace Solid-Phase Microextraction coupled with Gas Chromatography–Ion Mobility Spectrometry (HS-SPME/GC-IMS) [25]. The overall differences in volatile compounds between the two aquaculture systems were first visualized by means of principal component analysis (PCA). As shown in Figure 2A, the distinct separation between TPAS and RAS groups along the principal components indicates significant differences in their overall volatile compound profiles, which is consistent with the electronic nose results. Furthermore, the chord diagram (Figure 2B) intuitively illustrates the differences in the proportion of various volatile compound categories between the two groups. It clearly shows that the RAS group had a higher proportion of alcohols and esters, while the TPAS group had a higher proportion of aldehydes, providing a visual overview of the flavor composition differences.

Aldehydes—particularly nonanal, octanal, decanal, and heptanal—have been identified as the primary contributors to fishy odors [26]. Octanal and nonanal, oxidation products of oleic acid with low odor thresholds, impart fishy, fatty, and grassy notes, significantly influencing off-flavor formation [27]. Aldehyde content was significantly lower in the RAS group (12.82%) than in the TPAS group (16.13%), indicating that the RAS effectively reduces fishy odors. This may stem from the RAS influencing lipid metabolism, potentially inhibiting the generation of fishy odor-related VOCs [28]. The three-dimensional spectrum of GC-IMS (Figure 2C) provides a comprehensive visualization of the entire volatile compound profile [29], where each point represents a specific VOC. The differences in the position and intensity of the signal points between the two groups reflect the differences in the types and concentrations of VOCs. The overall signal intensity and distribution pattern in the RAS group differed from that of the TPAS group, confirming the fundamental impact of the aquaculture system on the volatile flavor profile of mandarin fish muscle. For a more precise comparison, the Gallery Plot (Figure 2D) was employed. This plot acts like a ‘fingerprint’ map, allowing for intuitive visual comparison of the differences in the content of each volatile compound between samples [30]. In the plot, each row represents a specific compound, and each column represents a sample group. It can be clearly observed that the signal intensities of many aldehydes (e.g., nonanal and heptanal) were higher in the TPAS group (darker color), whereas the signal intensities of several key alcohols (e.g., 1-octen-3-ol) and esters were more pronounced in the RAS group. This visual evidence strongly supports the quantitative findings that the RAS enhances desirable aromas and reduces off-flavors. Alcohol content, notably 1-octen-3-ol (imparting mushroom notes) [31], was significantly higher in the RAS (16.64% vs. TPAS: 14.77%). Enhanced lipid catabolism increases the levels of short-chain alcohols and enols, intensifying aroma [32]. Esters, formed via esterification and contributing floral notes [33], also increased significantly in the RAS, enhancing desirable flavors. Hydrocarbons, primarily derived from fatty acid homolytic cleavage, have minimal direct flavor impact due to high thresholds but may harmonize overall flavor [34]. Relative increases in alkenes, acids, and aromatics within the RAS further enriched the overall aroma profile. Collectively, these findings demonstrate that RAS rearing improves mandarin fish flavor quality by reducing fishy odors and enhancing desirable aroma compounds.

### 3.6. Gut Microbiome Analysis

#### 3.6.1. Gut Microbiota Diversity Analysis

**Figure 3:**
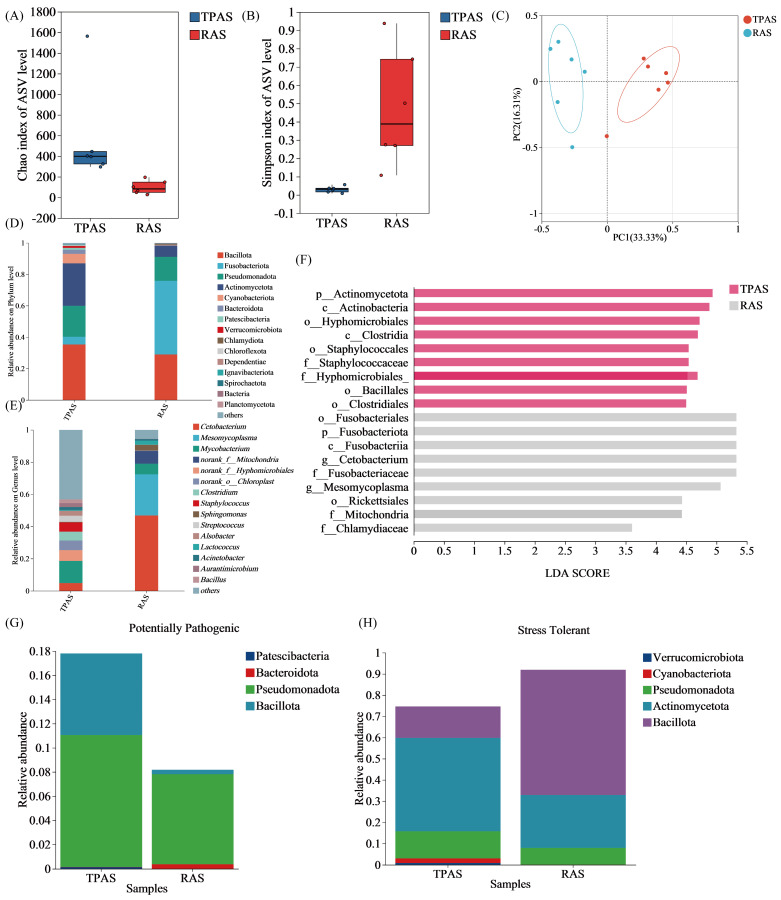
Chao1 (**A**), Simpson (**B**), PCA (**C**), relative abundance of the microbial community at the (**D**) phylum and (**E**) genus level, Lefse analysis: Histogram of LDA value distribution (**F**), phenotypic prediction based on BugBase analysis, potential pathogenicity (**G**), and stress tolerance (**H**).

Figure 3 shows the changes in alpha diversity of the intestinal microbiota of mandarin fish under the two aquaculture systems. Alpha diversity is generally used to analyze the microbial diversity within a single sample [35]. Among them, the Chao1 index and observed species index are parameters reflecting species richness. The Shannon index and Simpson index are parameters reflecting species diversity, and a larger Shannon index indicates higher diversity of community microorganisms. The Chao1 and Shannon indices show that the indices of the TPAS group are greater than those of the RAS group. The results of the Simpson diversity index indicate that the microbial diversity of the RAS group is significantly higher than that of the TPAS group (*p* < 0.05), suggesting that the RAS improved the alpha diversity of the intestinal microbiota of mandarin fish. Beta diversity analysis was performed on the intestinal microbiota of mandarin fish under the two aquaculture systems. Beta diversity is usually used to analyze the differences in microbial community structure between different samples [36]. It can be seen from the principal component analysis (PCA) plot in Figure 3A that the TPAS and RAS are clearly separated, with significant differences in community composition

#### 3.6.2. Gut Microbiota Taxonomic Composition Analysis

Figure 3 reveals the dominant microbial phyla in the intestinal flora of mandarin fish under the two aquaculture systems, including Firmicutes, Actinobacteria, Pseudomonadota, Fusobacteria, and Cyanobacteria, each accounting for different proportions within the microbial community. Most of these microbial groups are involved in the metabolism of carbohydrates, lipids, and amino acids, producing short-chain fatty acids (SCFAs) such as butyric acid, propionic acid, and acetic acid, thereby promoting energy utilization and nutrient absorption. Firmicutes and Bacteroidetes (particularly butyrate-producing groups) can produce various biologically active substances, including metabolites with antibacterial and antiviral effects, which help maintain intestinal ecological balance and enhance intestinal barrier function.

Compared to the TPAS group, the microbial community of the RAS group showed significant differences at multiple time points (LDA > 4.0, *p* < 0.05). Among them, Bacteroidetes, Actinobacteria, and Cyanobacteria were significantly enriched in the TPAS group (LDA > 4.0, *p* < 0.05). Bacteroidetes are commonly found in the intestines of fish, especially freshwater fish. They can decompose complex carbohydrates (such as cellulose) and proteins, playing a key role in the breakdown of polysaccharides and cellulose, thereby enhancing energy metabolism and immune regulation [37]. Although Bacteroidetes have a positive impact on intestinal health, their excessive abundance in the intestines may be associated with certain inflammatory diseases [38]. Therefore, the lower abundance of Bacteroidetes in the RAS group may contribute to improved fish health.

At the genus level, the relative abundances of *Mycobacterium*, *Clostridium*, and *Staphylococcus* in the intestinal flora of mandarin fish in the RAS group decreased, while the relative abundances of *Cetobacterium* and *Mesomycoplasma* increased. The results indicated that under the RAS, the relative abundances of potential pathogenic genera (such as *Mycobacterium*, pathogenic species of *Clostridium*, and *Staphylococcus*) in the intestines of mandarin fish were significantly reduced. This change is most likely directly related to the strict water quality management (e.g., efficient filtration and disinfection) and robust biosecurity measures in the RAS, which significantly reduce the pathogenic load and transmission risk in the environment, thereby effectively lowering the risk of mandarin fish contracting intractable bacterial diseases (such as mycobacteriosis) and intestinal or systemic diseases caused by opportunistic pathogens (such as pathogenic *Clostridium* and *Staphylococcus*). This is of critical importance for improving the health level and survival rate of fish. Simultaneously, the relative abundance of *Cetobacterium* in the intestines of mandarin fish under the RAS increased significantly. As a core symbiotic bacterium in the fish intestine, the increase in its abundance is particularly beneficial. *Cetobacterium* can efficiently ferment and produce SCFAs (such as acetic acid), providing additional energy for the host, enhancing intestinal barrier function, regulating local immune homeostasis (exerting anti-inflammatory effects), and potentially inhibiting the growth of some harmful bacteria [39]. This significantly promotes intestinal health and may improve feed utilization efficiency (especially adaptation to high-protein feed).

Certain bacterial species within Pseudomonadota, commonly referred to as pathogenic bacteria, may trigger chronic intestinal inflammation [40]. Pseudomonas aeruginosa is a typical opportunistic pathogen. In our study (Figure 3G), the relative abundance of Pseudomonadota in the intestinal microbial community of the TPAS group significantly increased, which may indicate the presence of a certain degree of intestinal inflammatory response in their bodies. Among stress-resistant microorganisms (Figure 3F), the abundance of Bacillota in the RAS group was significantly higher. Many bacteria of the genus *Lactobacillus* within this phylum are probiotics, which can regulate the homeostasis of the intestinal microbiota and contribute to maintaining the balance of the intestinal microecology, thereby potentially controlling various intestinal diseases and promoting overall health [41,42]. In general, the TPAS group had a higher relative abundance of conditional pathogens and a lower relative abundance of beneficial bacteria, indicating that the intestinal homeostasis and health of mandarin fish may have been damaged to a certain extent. On the contrary, the RAS group had a lower relative abundance of conditional pathogens and a higher relative abundance of beneficial bacteria, suggesting that this aquaculture model can effectively shape the intestinal flora and improve disease resistance.

#### 3.6.3. Gut Microbiota Function Analysis

**Figure 4:**
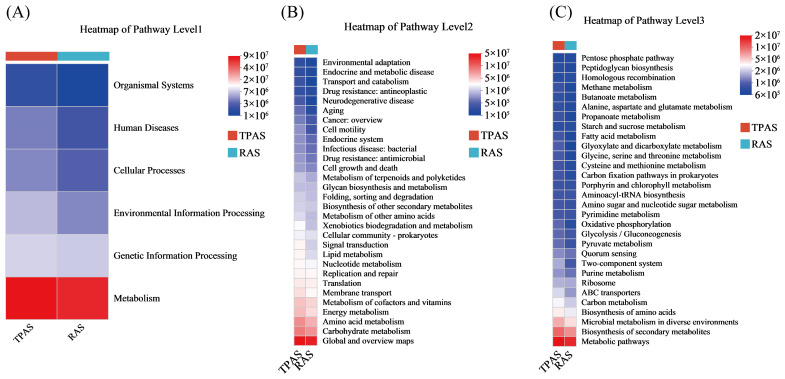
Microbial functional pathway heatmap: Level A (**A**), level B (**B**), and level C (**C**).

Figure 4 (Microbial Functional Pathway Heatmap) further reveals differences in the metabolic potential of the intestinal microbiota under the two aquaculture systems at the functional level. As shown in Figure 4A (Pathway Level1), both groups exhibited high activity in the “Metabolism” pathway, but the TPAS group showed more complexity in pathways related to “Human Diseases,” consistent with the potential mechanism of inflammation induced by opportunistic pathogens. The Level2 pathway heatmap indicated that the TPAS group was more active in stress- and disease-related pathways such as “Environmental Adaptation,” “Endocrine and Metabolic Diseases,” and “Antibiotic Resistance.” The Level3 pathway heatmap (Figure 4C) further verified that pathways such as “Biotin Metabolism” and “Porphyrin and Chlorophyll Metabolism” were enhanced in the TPAS group, reflecting the metabolic stress of microorganisms in response to inflammatory states. In contrast, the RAS group was enriched in probiotic-associated pathways such as “Lysine Biosynthesis” and “Carbon Metabolism,” which support nutrient absorption and microecological balance. In summary, the RAS significantly optimized the structure of the intestinal flora in mandarin fish through its advantages in environmental control: it inhibited the colonization and pathogenic risk of potential pathogens while promoting the proliferation of beneficial symbiotic bacteria represented by *Cetobacterium* and their metabolic functions (e.g., SCFA production). Combined with functional prediction analysis, the TPAS group exhibited characteristics of high abundance of opportunistic pathogens, low abundance of beneficial bacteria, and active disease-related functional pathways, which may impair intestinal homeostasis in mandarin fish to some extent. In contrast, the RAS group showed the opposite trend, with beneficial bacteria and healthy metabolic pathways dominating. This transformation in microbial structure and function serves as an important microbiological basis for the RAS to improve the health level of mandarin fish, reduce disease incidence, and enhance potential production performance. More importantly, the aforementioned changes in intestinal microbiota composition and function directly or indirectly influenced the muscle quality of mandarin fish through multiple metabolic pathways. The enrichment of beneficial bacteria in the RAS group and their involvement in pathways such as carbon metabolism and amino acid biosynthesis not only enhanced the host’s nutritional metabolism and energy utilization efficiency but also contributed to promoting protein deposition and the accumulation of flavor-related amino acids (e.g., Gly and Pro), while simultaneously inhibiting lipid peroxidation and the formation of fishy aldehydes. Furthermore, SCFAs (e.g., acetate), as key metabolites of microbial fermentation, may participate in systemic energy allocation and lipid metabolism via blood circulation, in addition to maintaining intestinal health. This further promotes higher crude protein content, lower crude fat content, and improved texture and flavor characteristics in the muscle.

### 3.7. Correlation Analysis

**Figure 5:**
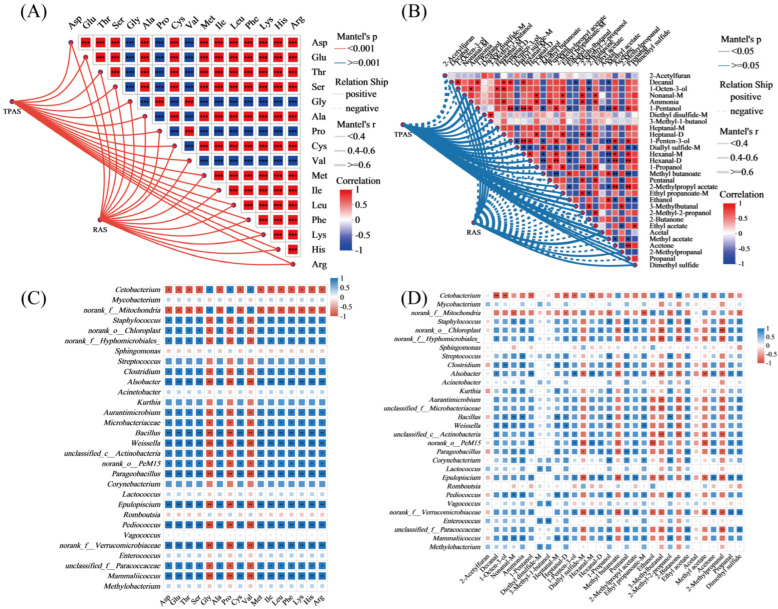
Mantel test network correlation heatmap of intestinal microbes with amino acids (**A**) and volatile flavor (**B**) substances in mandarin fish. Correlation between gut microbiota and free amino acid concentration (**C**) and correlation between gut microbiota and volatile flavor substance content (**D**). * *p* < 0.05, ** *p* < 0.01, and *** *p* < 0.001.

The Mantel test was employed to assess the correlation between intestinal microbiota composition and the profiles of amino acids (A) and volatile flavor compounds (B) in mandarin fish under two aquaculture systems. The analysis revealed significant associations between specific microbial taxa and muscle quality indicators. In the TPAS group (Figure 5A), a stronger positive correlation was observed between certain microbiota and bitter amino acids (e.g., His), alongside negative correlations with umami and sweet amino acids. Conversely, the RAS group exhibited a more favorable microbial composition, with positive correlations between beneficial genera (e.g., *Cetobacterium*) and key umami amino acids such as Glu and Asp, suggesting a potential role of these microbes in enhancing desirable flavor profiles. Regarding volatile compounds (Figure 5B), the RAS group showed significant negative correlations between specific microbiota and undesirable fishy odor compounds (e.g., nonanal and heptanal), supporting the earlier finding that the RAS reduces off-flavor components. Positive correlations were noted between certain microbes and pleasant aroma compounds such as esters and alcohols, further indicating that the RAS fosters a microbiota environment conducive to improved flavor quality. These results underscore the close relationship between gut microbiota structure and muscle quality traits, highlighting how the RAS modulates microbial communities to positively influence the nutritional and sensory attributes of mandarin fish.

Through further analysis of Figure 5C,D, we found that certain beneficial bacterial genera (such as *Cetobacterium*) in the RAS group showed a significant positive correlation with sweet amino acids (such as Gly and Pro) and pleasant flavor substances (such as esters and alcohols), while exhibiting a negative correlation with undesirable flavor precursors (such as certain aldehydes). This indicates that the RAS promotes metabolic pathways beneficial to flavor and nutrition by regulating the composition of intestinal microorganisms. In contrast, in the TPAS group, certain conditional pathogenic bacteria (such as *Staphylococcus* and *Acinetobacter*) were positively correlated with bitter amino acids (such as His) and fishy aldehydes (such as nonanal, heptanal), which further confirms the reason for its poor flavor quality. From the perspective of microbe–metabolite interaction, these results reveal the microbiological mechanism of the RAS in improving the muscle quality of mandarin fish.

## 4. Conclusions

This study comprehensively demonstrates that the recirculating aquaculture system (RAS) significantly enhances the overall muscle quality of mandarin fish (*Siniperca chuatsi*) compared to the TPAS. Specifically, the RAS effectively improved textural properties by reducing hardness and lowering adhesion, promoted a healthier nutritional profile with higher crude protein and lower crude fat content, and optimized flavor characteristics by increasing levels of key sweet-tasting amino acids (Gly and Pro) and desirable volatile compounds (e.g., esters and alcohols), while reducing off-flavor aldehydes (e.g., nonanal and heptanal). More importantly, gut microbiota analysis revealed that RAS cultivation increased microbial diversity and beneficial genera, notably *Cetobacterium* and *Lactobacillus*, which were positively correlated with flavor-enhancing amino acids and pleasant aroma compounds. Conversely, TPAS fish exhibited higher abundances of potential pathogens and inflammation-related metabolic pathways. The Mantel test further confirmed strong microbiota–flavor interactions, underscoring the role of the RAS in shaping a microbiome conducive to superior muscle quality. These findings provide mechanistic insights into how the RAS modulates gut microbial communities and metabolic pathways that are associated with improved muscle texture, nutrition, and flavor profile. However, it is important to acknowledge that the higher construction, operational costs, and technical demands of the RAS may limit its widespread adoption. Despite these economic considerations, the RAS enables year-round production independent of seasonal constraints and consistently yields superior product quality, as demonstrated in this study. Future studies should evaluate its economic viability and long-term stability. This study supports the adoption of the RAS as a sustainable and efficient aquaculture strategy for producing high-quality mandarin fish with enhanced organoleptic and nutritional value. This finding contributes to the sustainable and high-efficiency advancement of intensive mandarin fish aquaculture in China.

## Figures and Tables

**Table 1:** Comparison of muscle physical properties of mandarin fish in different culture systems.

Physical Characteristics	TPAS	RAS
Hardness	3044.14 ± 321.15 ^a^	2428.54 ± 125.61 ^b^
Adhesion	−24.55 ± 0.63 ^b^	−37.94 ± 1.56 ^a^
Elasticity	0.44 ± 0.06 ^a^	0.42 ± 0.08 ^a^
Mastication	612.61 ± 42.98 ^a^	514.37 ± 21.33 ^b^
Resilience	0.319 ± 0.05 ^a^	0.29 ± 0.06 ^a^

Values are expressed as the means ± S.D. Different letters in the same row indicate a significant difference (*p* < 0.05). TPAS: Traditional Pond Aquaculture System; RAS: Recirculating Aquaculture System.

**Table 2:** Comparison of muscle chemical compositions of mandarin fish in different culture systems.

Chemical Compositions	TPAS	RAS
Moisture (%)	79.13 ± 0.31 ^a^	78.49 ± 0.75 ^a^
Crude fat (%)	1.96 ± 0.01 ^a^	1.41 ± 0.01 ^b^
Crude ash (%)	1.20 ± 0.05 ^a^	1.17 ± 0.01 ^a^
Crude protein (%)	17.26 ± 0.26 ^b^	18.92 ± 0.15 ^a^

Values are expressed as the means ± S.D. Different letters in the same row indicate a significant difference (*p* < 0.05). TPAS: Traditional Pond Aquaculture System; RAS: Recirculating Aquaculture System.

**Table 3:** Comparison of amino acid contents of mandarin fish muscle in different culture systems.

Amino Acid (mg/g)	TPAS	RAS
Asp	6.07 ± 0.97 ^a^	4.09 ± 0.76 ^b^
Glu	35.75 ± 1.09 ^a^	30.30 ± 2.44 ^b^
Thr *	38.06 ± 1.10 ^a^	31.53 ± 2.80 ^b^
Ser	83.34 ± 1.41 ^a^	43.89 ± 3.44 ^b^
Gly	69.84 ± 1.05 ^a^	108.39 ± 8.67 ^b^
Ala	58.18 ± 1.03 ^a^	36.67 ± 3.05 ^b^
Pro	59.43 ± 3.10 ^a^	107.75 ± 6.14 ^b^
Cys	1.43 ± 0.16 ^a^	0.89 ± 0.57 ^a^
Val *	12.85 ± 0.44 ^a^	13.85 ± 1.13 ^a^
Met *	3.14 ± 0.38 ^a^	2.63 ± 0.31 ^a^
Ile *	3.90 ± 0.27 ^a^	2.16 ± 0.51 ^b^
Leu *	5.47 ± 0.46 ^a^	3.80 ± 0.41 ^b^
Phe *	11.14 ± 0.29 ^a^	10.95 ± 0.37 ^a^
Lys *	38.77 ± 2.62 ^a^	31.99 ± 2.50 ^b^
His	51.11 ± 1.10 ^a^	31.85 ± 0.80 ^b^
Arg	2.72 ± 0.15 ^a^	2.48 ± 0.32 ^a^
Umami amino acids	41.82 ± 1.91 ^a^	34.39 ± 3.10 ^b^
Sweet amino acids	308.85 ± 2.49 ^a^	328.23 ± 2.39 ^a^
Bitter amino acids	130.54 ± 3.66 ^a^	100.61 ± 4.46 ^b^
EAA	113.34 ± 2.92 ^a^	96.92 ± 6.93 ^b^
NEAA	367.87 ± 3.33 ^a^	366.31 ± 2.48 ^a^
Total amino acid	481.21 ± 5.49 ^a^	463.23 ± 3.12 ^a^
EAA/TAA%	0.24 ± 0.00 ^a^	0.21 ± 0.00 ^b^
EAA/NEAA%	0.31 ± 0.01 ^a^	0.26 ± 0.01 ^b^

Values are expressed as the means ± S.D, * indicates essential amino acids. Different letters in the same row indicate a significant difference (*p* < 0.05). TPAS: Traditional Pond Aquaculture System; RAS: Recirculating Aquaculture System.

## Data Availability

The original contributions presented in this study are included in the article/Supplementary Material. Further inquiries can be directed to the corresponding authors.

## Supplementary Materials

The following supporting information can be downloaded at https://www.mdpi.com/article/10.3390/foods14234028/s1. Table S1: Identification of volatile compounds and their odor characteristics; Table S2: Differential taxa identified by LEfSe analysis.
